# Morphological and Genomic Features of the New Klosneuvirinae Isolate Fadolivirus IHUMI-VV54

**DOI:** 10.3389/fmicb.2021.719703

**Published:** 2021-09-21

**Authors:** Julien Andreani, Frederik Schulz, Fabrizio Di Pinto, Anthony Levasseur, Tanja Woyke, Bernard La Scola

**Affiliations:** ^1^Aix-Marseille Université, IRD, APHM, MEPHI, IHU Méditerranée Infection, Marseille, France; ^2^DOE Joint Genome Institute, Lawrence Berkeley National Laboratory, Berkeley, CA, United States

**Keywords:** Vermamoeba vermiformis, giant virus, translation components, Mimiviridae, ATPase subunits, Klosneuvirinae, Fadolivirus

## Abstract

Since the discovery of *Mimivirus*, viruses with large genomes encoding components of the translation machinery and other cellular processes have been described as belonging to the nucleocytoplasmic large DNA viruses. Recently, genome-resolved metagenomics led to the discovery of more than 40 viruses that have been grouped together in a proposed viral subfamily named *Klosneuvirinae*. Members of this group had genomes of up to 2.4Mb in size and featured an expanded array of translation system genes. Yet, despite the large diversity of the Klosneuvirinae in metagenomic data, there are currently only two isolates available. Here, we report the isolation of a novel giant virus known as Fadolivirus from an Algerian sewage site and provide morphological data throughout its replication cycle in amoeba and a detailed genomic characterization. The Fadolivirus genome, which is more than 1.5Mb in size, encodes 1,452 predicted proteins and phylogenetic analyses place this viral isolate as a near relative of the metagenome assembled Klosneuvirus and Indivirus. The genome encodes for 66 tRNAs, 23 aminoacyl-tRNA synthetases and a wide range of transcription factors, surpassing Klosneuvirus and other giant viruses. The Fadolivirus genome also encodes putative vacuolar-type proton pumps with the domains D and A, potentially constituting a virus-derived system for energy generation. The successful isolation of Fadolivirus will enable future hypothesis-driven experimental studies providing deeper insights into the biology of the *Klosneuvirinae*.

## Introduction

In 2003, with the first description of Mimivirus (La Scola, 2003), viruses were revealed to be more complex entities than previously thought and with extraordinary properties, e.g., numerous tRNAs, aminoacyl-tRNA synthetases (aaRS) and themselves being infected by virophages (La Scola et al., 2008; Gaia et al., 2014). In terms of capsid size, as well as gene content, this first discovery created a paradigm shift and shattered extant viral definition (Forterre, 2016; Colson et al., 2017). 18years later, giant viruses with ovoid forms, even larger genome sizes, and viruses with no capsid have been discovered with the successive isolations of Pandoraviruses (Philippe et al., 2013; Antwerpen et al., 2015; Aherfi et al., 2018; Legendre et al., 2018), Pithoviruses (Legendre et al., 2014; Levasseur et al., 2016), Cedratviruses (Andreani et al., 2016; Bertelli et al., 2017; Silva et al., 2018; Rodrigues et al., 2018a; Boudjemaa et al., 2020), and Orpheovirus (Andreani et al., 2018). In addition, the description of Meelsvirus revealed that these potential viruses can take extremely varied forms and are associated with a complex formation of their capsids (Shinn and Bullard, 2018). These large viruses share a common origin and a novel name has been proposed: Nucleocytoviricota (replacing the term nucleocytoplasmic large DNA viruses (NCLDV; Koonin et al., 2020).

In parallel, advances in (meta) genomic sequencing of viruses expanded rapidly (Verneau et al., 2016; Schulz et al., 2017, 2018, 2020; Wilson et al., 2017; Moniruzzaman et al., 2020a,b). In 2017, a metagenomic study reported the detection and genome reconstruction of four new viruses affiliated with the Mimiviridae which encoded expanded protein biosynthesis components, Klosneuvirus, Indivirus, Catovirus, and Hokovirus, proposed as a sub-family, *Klosneuvirinae* (Schulz et al., 2017). Just 1year later, the diversity of this unexplored viral group was further expanded with the discovery of seven additional metagenome assembled viruses from soil (Schulz et al., 2018) and the first isolate affiliated with the Klosneuvirinae, Bodo saltans virus (Deeg et al., 2018). In contrast to many other members of the Klosneuvirinae, Bodo saltans virus encodes for only two functional aminoacyl-tRNA synthetases (aaRS) and no tRNA. In the meantime, two novel members of the Mimiviridae were isolated, Tupanvirus soda lake and Tupanvirus deep ocean, possessing 70 tRNA and 20 aaRS, and similar and slightly more complete translational components than the metagenomic Klosneuvirus (Abrahão et al., 2018). Tupanviruses do not group with the Klosneuvirinae but with the Megamimivirinae, sharing a hypothetical ancestor with the three lineages of Mimiviruses A, B, and C (Rodrigues et al., 2018b). The first isolate of the Klosneuvirinae that had an expanded set of translation system genes was the icosahedral virus in *Vermamoeba vermiformis,* named Yasminevirus (Bajrai et al., 2019). Its genome is one of the largest in the Mimiviridae with 2.1Mb and possesses various components of a complete translation system. The discovery of these viruses has opened up new perspectives regarding their evolution in relation to eukaryotes. Recently, metagenomic has revealed the widespread presence of energy metabolism encoded in the Nucleocytoviricota, such as photosynthesis genes and enzymes playing roles in glycolysis among others (Schulz et al., 2020; Moniruzzaman et al., 2020a).

In this study, we report the isolation and detailed description of the closest relative of the metagenomic Klosneuvirus from an Algerian sewage sample, representing the third viral isolate of the *Klosneuvirinae*. Our study provides information on the viral replicative cycle and host range, as well as its phylogeny and genomic features, revealing a more complete picture of the *Klosneuvirinae*. Our isolate contributes toward establishing an experimental framework of viral strains that may be used to study the biology of this diverse subfamily of the Mimiviridae.

## Materials and Methods

### Sample Collection and Virus Isolation

Samples from which the virus was isolated were part of a study aiming to evaluate giant virus diversity in north-west Algeria (Boudjemaa et al., 2020). Briefly, a sample was collected from sewage in Sidi Bel Abbès (Algeria) on the September 1, 2017. As previously reported (Geballa-Koukoulas et al., 2020, 2021), we used *Vermamoeba vermiformis* stain CDC19 as cell support. The amoebae were harvested after 48h of culture in peptone yeast extract glucose medium (Eurobio, France) when a concentration of 1.10^6^ amoebae/mL was reached. Cells were then rinsed twice in homemade Page’s Amoeba Saline and pelleted at 700×g for 10min. The amoebae were then resuspended in the starvation medium (Reteno et al., 2015). A cell suspension of 500μl per well was distributed onto a 24-well plate. The samples were vortexed, and 50μl was added to each well. Remaining wells served as negative controls. The plate was incubated at 30°C for 4days in order to monitor any potential cytopathic effect. This co-culture was repeated twice in the same order.

### Host Range Survey

Multiple hosts were tested by inoculation of Fadolivirus on *Vermamoeba vermiformis* (ATCC^®^ 50237, initial support of isolation), *Vermamoeba vermiformis* strain chuv172, *Acanthamoeba castellanii* strain Neff (ATCC^®^ 30010), *Acanthamoeba polyphaga* strain Linc-AP1, *Acanthamoeba royreba* strain Oak ridge (ATCC^®^ 30884), *Disctyostelium discoideum* (ATCC^®^ 44841), the flagellated alga *Tetraselmis* sp. (IHU isolate), and the protist *Cafeteria roenbergensis* (IHU isolate). The lytic events were monitored by observations under an inverted microscope, as described for Tupanvirus (Abrahão et al., 2018), for 5days.

### Flow Cytometry Analyses

According to previously described protocols (Khalil et al., 2016; Andreani et al., 2017), we centrifuged the viral supernatant at 700× *g* for 10min, added a 1:100 SYBR green dye dilution (SYBR green I nucleic acid gel stain; Molecular Probes, Life Technologies), and then heated the mixture at 80°C for 3min. Stained viral populations were acquired on a BD LSR Fortessa (BD Biosciences) cytometer and compared with previously recorded gates for known viral isolates on FlowJo software.

### Electron Microscopy Observations

We used protocols similar to previous studies (Andreani et al., 2016, 2017) for embedding and transmission electron microscopy. The only difference was the infection time points at which the samples were collected. The time points were 0, 4, 8, 12, 16, 20, 24, and 36h post-infection. Microscopy was performed using a Tecnai G20 electron microscope (FEI, Germany) operating at 200keV, and the size of the particles was measured using ImageJ.^1^

### Viral Production and Purity Control

We successively inoculated diluted viral supernatant on *V. vermiformis* at a dilution factor of 10 in order to clone the virus before its production. End point dilution was assessed for 5days and the lysis was controlled by inverted microscopy. For the production and purification processes, 15 flasks of 150cm^2^ (Corning^®^, Corning, NY, United States) were infected. After 4days, we filtrated all the supernatants from flasks using 0.8μm filters. The filtrate supernatant was then pelleted using the Beckman coulter^®^ Optima^™^ XPN-80 - IVD (Beckman, France) at 50,000×g for 30min. A 25% sucrose gradient was used for the final purification step. After finalizing production, we extracted DNA by using the EZ1 Advanced XL device (Qiagen, Courtaboeuf, France).

### Genome Sequencing and Genome Assembly

Genomic DNA of Fadolivirus was sequenced on the MiSeq platform (Illumina Inc., San Diego, CA, United States) using a paired end strategy. In order to pool the sample with 18 other genomic projects for sequencing, a barcoded library was created using the Nextera XT DNA sample prep kit (Illumina). In detail, Fadolivirus gDNA was quantified using a Qubit assay using the high sensitivity kit (Life technologies, Carlsbad, CA, United States). DNA concentration was 5.7ng/μl. To prepare the paired end library, the gDNA was diluted to provide 1ng of input. The “tagmentation” step fragmented and tagged the DNA. A limited cycle PCR amplification (12cycles) completed the tag adapters and introduced dual-index barcodes. After purification on AMPure XP beads (Beckman Coulter Inc., Fullerton, CA, United States, the libraries were normalized on specific beads according to the Nextera XT protocol (Illumina). Normalized barcoded libraries were then pooled into a single library for sequencing on the MiSeq. The pooled single strand library was loaded onto the reagent cartridge and the instrument along with the flow cell. Automated cluster generation and paired end sequencing with dual index reads were performed in a single 39-h run in 2×250-bp. A total of 8.5Gb of Fadolivirus genomic sequence was obtained from a 927,000 cluster density per mm^2^ with a cluster passing quality control filters of 94.1% (16,738,000 clusters). Within this run, the index representation for this virus was determined to represent 6.8%. To improve the Fadolivirus assembly, a MinIon Oxford Nanopore run was performed.

The Oxford Nanopore approach was performed on 1D genomic DNA sequencing using the MinIon device and SQK-LSK108 kit. The sequencing library was constructed using 1.5μg genomic DNA previously extracted without fragmentation and end repair. Adapters were ligated to both ends of genomic DNA. After purification on AMPure XP beads (Beckman Coulter Inc., Fullerton, CA, United States), the library was quantified by a Qubit assay with the high sensitivity kit (Life technologies, Carlsbad, CA, United States). Approximately 20ng of the tethered library was loaded on the flow cell *via* the SpotON port. 317 active pores were detected for sequencing and the WIMP workflow was chosen for live bioinformatics analysis. After 36h of run time and end life of the flow cell, 13,136 raw reads were generated. The software EPI2ME lead to 13,122 analyzed viral sequence reads totaling 30.3Mb, with an average length of 2,305kb and a maximum read length of 32,328bp.

Paired-end Illumina reads and Minion reads were *de novo* assembled using the hybridSPAdes algorithm (Antipov et al., 2016). We obtained two scaffolds representing a total size of 1,595,395bp with an average read coverage of 121-fold for the first scaffold and 102-fold for the second.

### Genome Analysis

Gene predictions were computed using Prodigal (Hyatt et al., 2010). We deleted 49 predicted proteins of fewer than 50 amino acids, or which had abnormal tri-dimensional folding (from 50 to 99 amino acids), as detected by Phyre2 (Kelley et al., 2015). Protein blast was performed against the non-redundant (nr) protein database. Annotation was performed using a combination of Interpro https://www.ebi.ac.uk/interpro/search/sequence-search version 69.0, a CD-search tool online (Marchler-Bauer and Bryant, 2004) and delta-blastp (Boratyn et al., 2012). tRNA prediction was computed online (Lowe and Chan, 2016) with the eukaryotic model parameter.

### Comparative Genomics

Orthofinder (v2.4; Emms and Kelly, 2015) was used to infer orthogroups from published genomes of representatives of all established Mimiviridae lineages and estimated high-quality giant virus metagenomes assembled genomes affiliated with the Klosneuvirinae. Further, Interproscan (v5.0; Jones et al., 2014) was employed to assign PFAM-A domains (v33.0; El-Gebali et al., 2019) to giant virus proteins. GenoplotR (Guy et al., 2010) was used to visualize genome synteny of conserved blocks that were inferred with Mauve (v2.4; Darling et al., 2004) in pairwise comparisons between genomes of selected members of the Mimiviridae and Fadolivirus.

### Genome Deposition Into Data Repository

The two scaffolds of Fadolivirus IHUMI-VV54 are available on the NCBI website under accession numbers MT418680.1 and MT418681.1.

### Phylogenetic Analysis—Species Tree

To infer the phylogenetic position of Fadolivirus in the Mimiviridae, five conserved NCLDV proteins (Yutin et al., 2009) were selected: DNA polymerase elongation subunit family B (NCVOG0038), D5-like helicase-primase (NCVOG0023), packaging ATPase (NCVOG0249), and DNA or RNA helicases of superfamily II (NCVOG0076) and Poxvirus Late Transcription Factor VLTF3-like (NCVOG0262), and identified with hmmsearch (version 3.1b2, hmmer.org). Protein sequences were extracted and aligned using mafft-lins (Katoh and Standley, 2016). Gapped columns in alignments (<10% sequence information) and columns with low information content were removed from the alignment with trimal (v1.4; Capella-Gutiérrez et al., 2009). Protein alignments were then concatenated to a supermatrix and a species tree of the Mimiviridae was built with IQ-tree (v2.03) with LG + F + R6 (Nguyen et al., 2015). The species tree was then visualized using iTOL v6 online (Letunic and Bork, 2016).

### Phylogenetic Analysis - Protein Trees

Phylogenetic analyses for ATPases were conducted as follows. Blastp was used to find 39 close homologous proteins, and amino acid sequences were then aligned using the MUSCLE program (Edgar, 2004). The Mega 6.0 software was used with standard parameters using the maximum likelihood method with 1,000 bootstrap replicates and the Jones–Taylor–Thornton model for amino acid substitution. Phylogenetic trees were then visualized using iTOL v6 online (Letunic and Bork, 2016).

## Results

### Isolation of a Novel Giant Virus and Host Range Test

**Figure 1:**
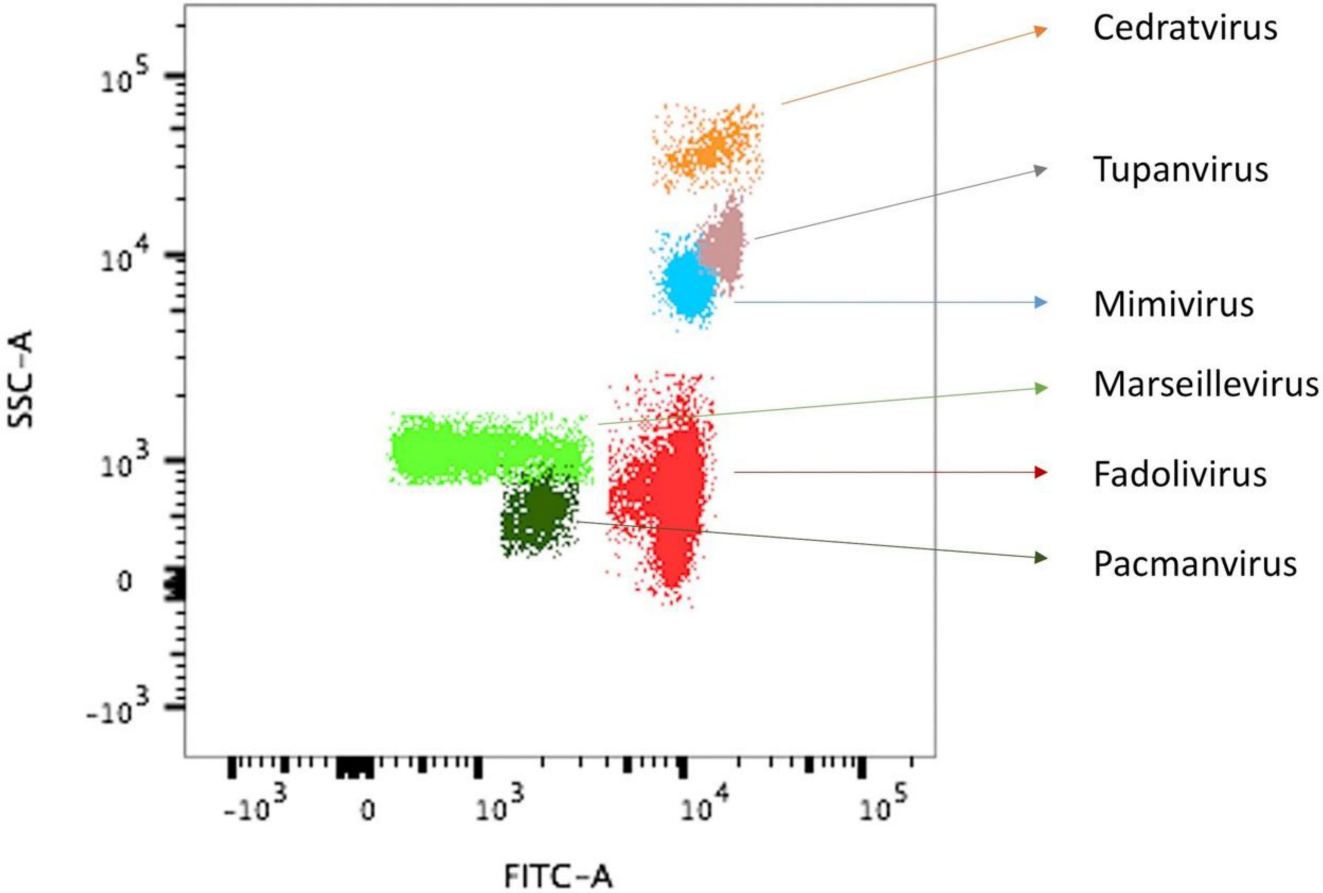
Observation of virus isolate VV54 sample on flow cytometry superimposed with that of other known viral isolates. Flow cytometry dot plot showing different viral profiles, in SSC (side scatter) *versus* FITC (Fluorescein isothiocyanate) SYBR green DNA contents, distinguishable at the levels of capsid shape (optical properties) and DNA components. For the sample VV54, the single viral population (Fadolivirus) is shown in red. For comparison, gates from previous isolates of other viral strains are shown.

Co-culture steps were performed on 64 samples on the amoeba *Vermamoeba vermiformis* strain CDC19 (Boudjemaa et al., 2020). Cell lysis was observed using an inverted optical microscope, with obvious lysis in well 54. Flow cytometry allowed us to detect a single novel population in our gating strategy (Khalil et al., 2016), with a high value of fluorescence compared to the side scatter fluorescence (Figure 1). We tried to infect various potential alternate hosts with this virus, specifically *Vermamoeba vermiformis* strain chuv172*, Acanthamoeba castellanii* strain Neff, *Acanthamoeba polyphaga* strain Linc-AP1, *Acanthamoeba royreba* strain Oak ridge, *Dictyostelium discoideum*, the flagellated alga *Tetraselmis* sp., and the protist *Cafeteria roenbergensis*. Fadolivirus was only able to infect *Vermamoeba vermiformis* strain CDC19, suggesting a narrow host range. No cytopathic effects were observed using optical microscopy for any of these potential hosts.

### Ultrastructure of Fadolivirus and Replication Cycle

**Figure 2:**
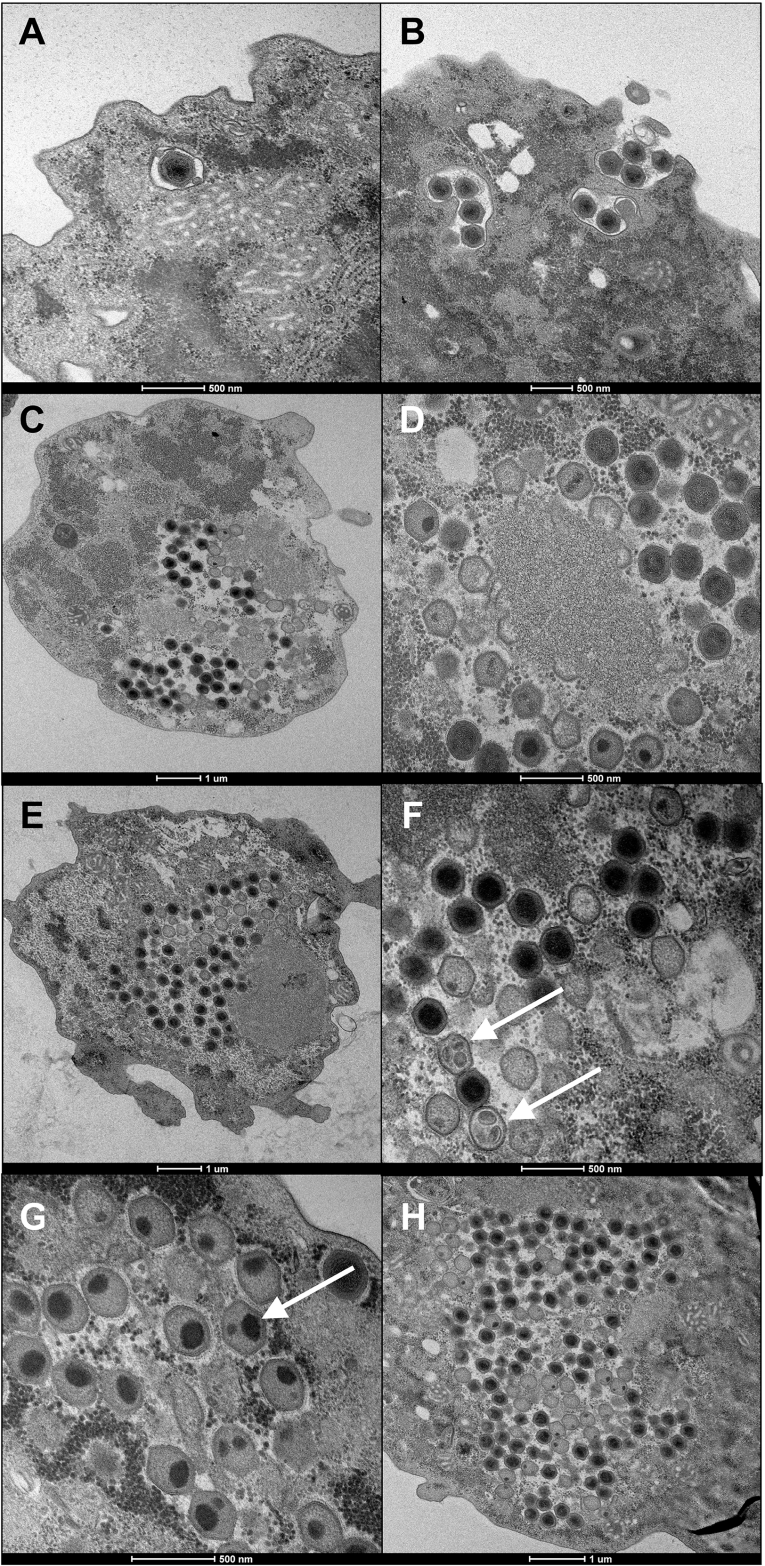
Ultrathin section of the replication cycle of Fadolivirus IHUMI-VV54. Scale bars are indicated under each panel. (**A, B**) were observed during viral entry at 0h and two hours post-infection. (**C**) Represents a section of *Vermamoeba vermiformis* 12h post-infection. (**D**) High magnification of a viral factory 16h post-infection. (E) New virus produced 20h post-infection. (**F, G**) White arrows indicate icosahedral capsid with ovoid points intra-capsidic localization. (**H**) Intra-cytoplasmic viruses observed 24h post-infection.

Fadolivirus particles are icosahedral with a diameter of about 300nm (*n*=21) on ultrathin sections. Multiple layers of high-density electrons (proteinaceous) were visible comparable to those described for Mimivirus, Yasminevirus and Bodo saltans virus (Deeg et al., 2018; Bajrai et al., 2019). The replication cycle of Fadolivirus showed classical NCLDV stages of infection and replication in *V. vermiformis* and small-sized fibrils were sometimes distinguishable in the periphery of the viral particles, as observed in Yasminevirus. Briefly, the replication cycle begins with the virus entry by phagocytosis. Next, particles escape the phagosomal process. DNA delivery occurs in the amoeba cytoplasm *via* the apex of the icosahedral virus (Figures 2A,B). After cytoplasmic liberation of the virus, an eclipse phase is observed. After 12h, viral factories are assembled within some amoeba (Figure 2C) and 16h post-infection (Figure 2D), nearly all cells present a well-established viral factory. While the Fadolivirus factory resembles that described for Mimivirus (Suzan-Monti et al., 2007), we found a singular element in the filling of the capsid. Indeed, after the synthesis of the icosahedral structure by the factory, the pseudo-ovoidal structure becomes increasingly dense and round inside this neo-formed capsid (Figures 2F,G). This step seems to be different compared to observations in Mimivirus. Finally, this gives the particle its mature aspect with a highly dense core. At 20h post-infection (Figure 2E) and 24h post-infection (Figure 2H), the host cells’ cytoplasm is fully occupied by newly synthesized virions. Cell burst begins 24h post-infection and is completed 36h after the viral infection.

### Genomic Features of Fadolivirus

**Figure 3:**
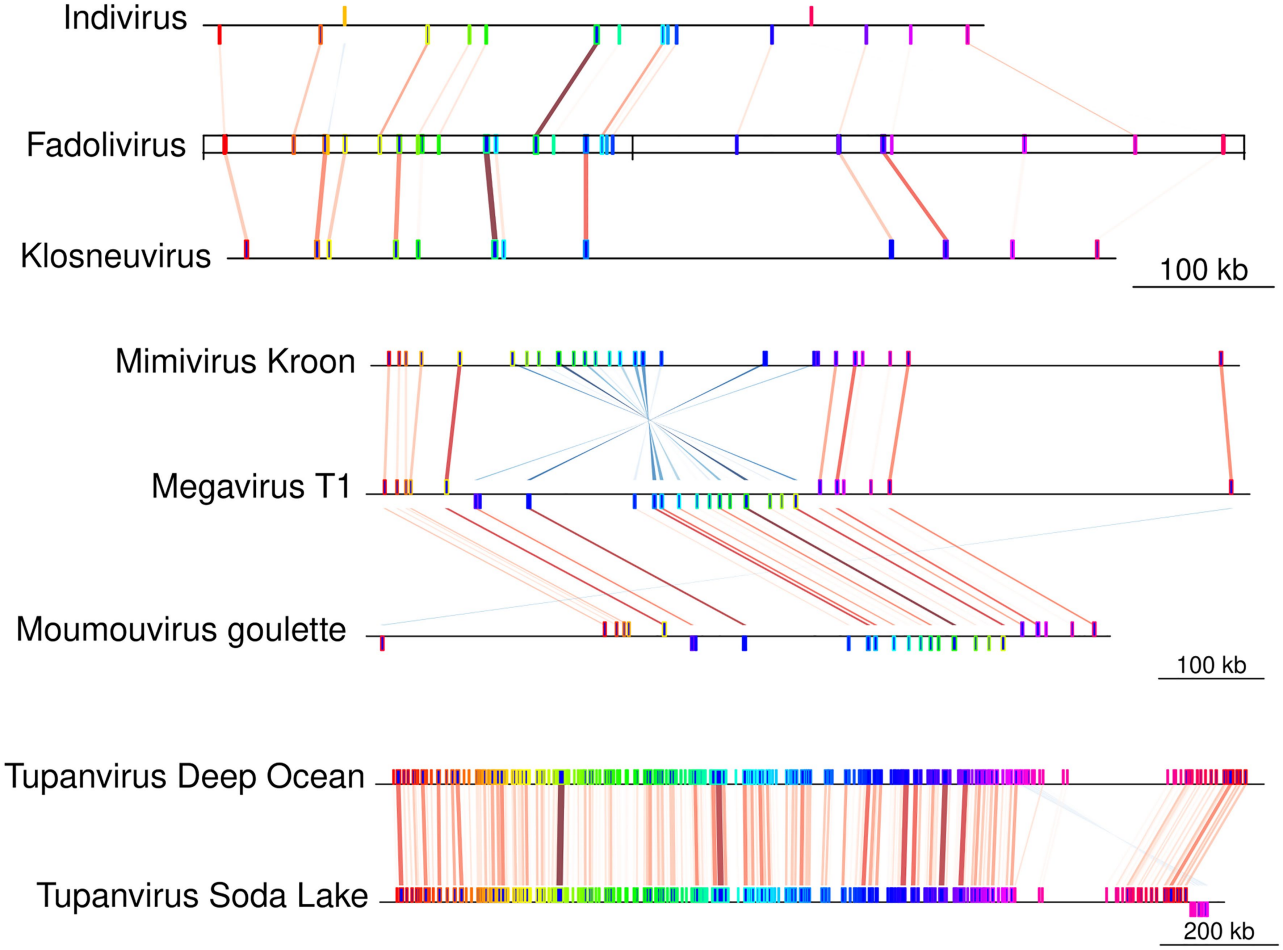
Block synteny comparison between Fadolivirus and other Klosneuvirinae genomes. Conserved genomic regions (Mauve conserved blocks) between Fadolivirus and its closest relatives, Klosneuvirus and Indivirus. Contigs in Indivirus and Klosneuvirus have been reordered based on the Fadolivirus genome. For comparison, conserved regions are also shown between selected members of the proposed subfamily Megamimivirinae, including Tupanviruses.

The Fadolivirus genome assembled into two scaffolds with a total length of 1,595,395bp (with 1,573,504bp for scaffold 1 and 21,891bp for scaffold 2; Table 1), encoding 1,452 genes of which 1,428 were on the larger scaffold, here referred to as the major scaffold. A megablast of the major scaffold against the nucleotide collection in the NCBI database revealed matches to Indivirus, Klosneuvirus, Catovirus, and Tupanviruses. A whole genome alignment showed that numerous blocks were conserved between Fadolivirus, Klosneuvirus KNV1 and Indivirus ILV1 (Figure 3). However, when aligned to the genome of Bodo saltans virus, the proportion of shared sequences decreased notably (data not shown).

**Table 1:** Morphological and genome characteristics of members of selected Klosneuvirinae and Tupanviruses.

Virus	Fadolivirus IHUMI	Yasminevirus	Klosneuvirus	Catovirus	Hokovirus	Indivirus	Bodo saltans virus	Tupanvirus soda lake	Tupanvirus Deep ocean
Morphological features	Icosahedral capsid	Icosahedral capsid	Unk	Unk	Unk	Unk	Icosahedral capsid	Icosahedral capsid with a tail	Icosahedral capsid with a tail
Genome size (Mbp)	1.59	2.12	1.57	1.53	1.33	0.86	1.38	1.43	1.51
GC content (%)	27.10	40.2	28.6	26.4	21.4	26.6	25.3	29.4	29.1
tRNA	66	70	25	3	0	1	0	67	70
Scaffolds	2	2	16	2	5	18	1	1	1
CDS	1,452	1,541	1,545	1,427	1,022	744	1,207	1,276	1,359
aaRS	23^*^	20	19	15	3	10	(3^*^+2)	20	20
CP	11	5	9	Und	Und	Und	4	3	3

*Unk, Unknown; CP, capsid protein; Und, Undetermined. homologous proteins of capsid proteins*.

* *3 in Bodo saltans virus, three aaRS are suggested to represent pseudogenes*.

* *a Glutaminyl-tRNA synthetase is split into two proteins by an HNH endonuclease*.

A blastp comparison of Fadolivirus proteins to the NCBI nonredundant database (nr) revealed the presence of 256 ORFans without any matches in nr (≈17,6% of all predicted proteins), 552 hits with Klosneuvirus KNV1 (≈38% of all predicted proteins), 223 with Indivirus ILV1 (≈15,4% of all predicted proteins), 24 with Catovirus CTV1, 21 with Hokovirus HKV1 and 24 with Tupanvirus strain deep ocean, 23 with Tupanvirus soda lake, and only 59 with other viruses. We also obtained 111 best hits to eukaryotic and 151 to bacterial and archaeal proteins. Based on the results of this analysis, it becomes evident that the gene content of Fadolivirus is most similar to that of Klosneuvirus. The Fadolivirus genome encodes 66 tRNAs that belong to 19 different types and represent 35 different anticodons (Table 1; Supplementary Material S1). Twenty-five were described for the Klosneuvirus KNV1 genome and only one for Indivirus ILV1 (Table 1). Similar to what has been described for other Klosneuvirinae (Schulz et al., 2017, 2018), Fadolivirus encoded a comprehensive set of translation system components consisting of various translation initiation factors (eIF4G, F, eIF-5A, B for example), elongation factors (aef-2), and a termination factor (Rho domain).

As previously observed in members of the Klosneuvirinae and in particular in Bodo saltans virus, there were numerous gene duplication events (102 clusters with than two proteins) in the Fadolivirus genome of which only 18 clusters had predicted functions. Thirteen of the duplicated genes were homologous to the capsid protein. More detailed analyses revealed that two ORFs encoded a putative major capsid protein (MCP) split by 35 nucleotides and six others were predicted to represent other MCP (Supplementary Material S1). The in-depth *in silico* analysis further led to the exclusion of a putative capsid which was predicted as a probable Zinc finger (NCBI QKF94282.1) domain containing protein. No other *Klosneuvirinae* genome encodes that many (11) capsid proteins. Other conserved NCLDV hallmark genes (Koonin and Yutin, 2010) were also present in multiple copies in the Fadolivirus genome, including the protein predicted to encode the VV-D5 helicase homologues and the A32 packaging ATPase and mRNA capping enzyme with three and four copies, respectively.

In contrast to other members of the *Klosneuvirinae*, Fadolivirus encodes proteins that may play a role in modulation of host cell processes (as for BAG domain-containing protein), vesicular transport as the soluble N-ethylmaleimide-sensitive factor (NSF), pyrimidine metabolism (dihydroorotate dehydrogenase 1A, orotidine 5′-phosphate decarboxylase), transcription and catabolism (putative transcriptional regulator ICP4, regulator of protease activity HflC), in the vitamin K metabolism (vitamin K epoxide reductase and vitamin K dependent gamma-carboxylases (VKGC)), efflux protein (small multidrug resistance protein (multidrug transporter EmrE), in the channel formation (major intrinsic protein, and two predicted proteins in the peptidoglycan synthesis (D-ala-D-ala ligase).

We also retrieved a V21 virophage-like protein (initially described in the Sputnik virophage) that had recently been suggested to play a similar function to transcription factor, as found in other giant virus genomes such as Orpheovirus IHUMI-LCC2 (Andreani et al., 2018). However, no virophage was isolated together with Fadolivirus. Fadolivirus encodes a homolog of Cyclophilin, a chaperone with a role in protein folding (Barik, 2018), yet its role in the Fadolivirus biology remains currently unclear. In the *Acanthamoeba polyphaga Mimivirus*, phosphodiesterase activity was not found (Thai et al., 2008). The Fadolivirus genome also contains three genes annotated as cytochromes (one as 5b, two as P450) and two probable lactonases. Cytochrome has recently been shown to have a broad distribution in DNA viruses and may be involved in cholesterol/sterol synthesis (Lamb et al., 2019).

### Fadolivirus Genes Involved in Energy Metabolism

**Figure 4:**
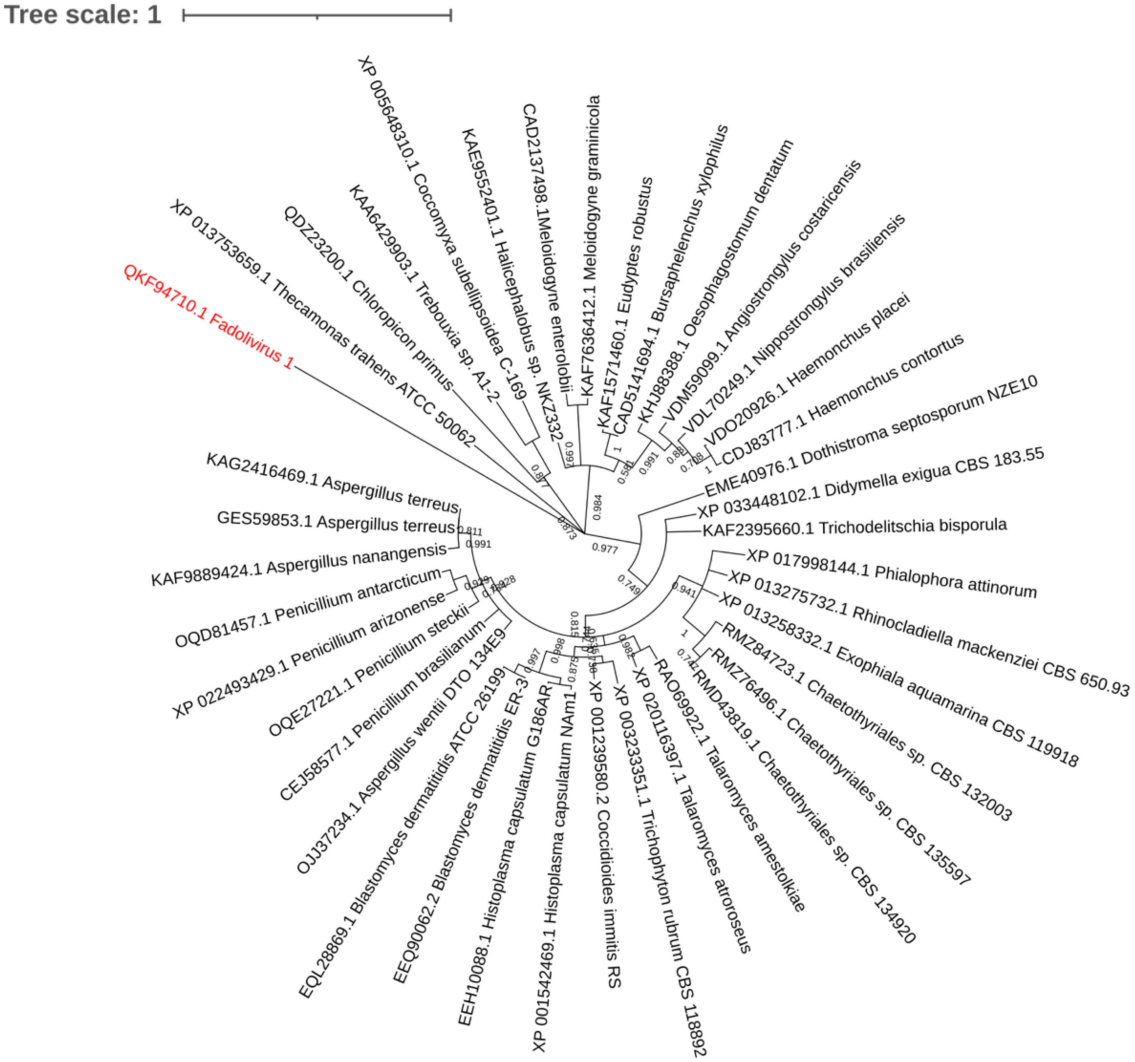
Maximum-likelihood representation built with the Jones–Taylor–Thornton method with 1,000 bootstrap replication based on 286 aligned positions. 39 eukaryote sequences were downloaded as best hit with ATPase from Fadolivirus.

The Fadolivirus genome annotation revealed multiple sequences annotated as putative ATPase subunits and proteins associated with electron transfer with a blue copper enzyme and a putative cytochrome 5b (see previous section). This cytochrome sequence is a probable nitrate reductase (NADH) protein (Chamizo-Ampudia et al., 2017) in the Fadolivirus genome. We identified six proteins associated with proton pumps containing ATPase domains. However, these genes were not co-located in the genome of Fadolivirus. A more detailed phyre2 analysis of these 6 proteins revealed that two of them were indeed predicted as v-type proton ATPase subunit D (QKF94710.1) with 100% confidence and 28% identity with the Yeast V-ATPase state 3 (PDB database 3J9V), and with the other states 1 and 2. This protein could be a vacuolar proton pump hydrolyzing the ATP to ADP (Balakrishna et al., 2015; Zhao et al., 2015). For the protein QKF93735.1, it presents known homologies with vacuolar ATP synthase subunit A by DELTA-blast. This potential subunit A is shared with other giant viruses in Klosneuvirinae, and the predicted subunit D is divergent compared to the eukaryotes (Figure 4).

### Genomic Diversity Across Klosneuvirinae and Phylogenetic Position of Fadolivirus

**Figure 5:**
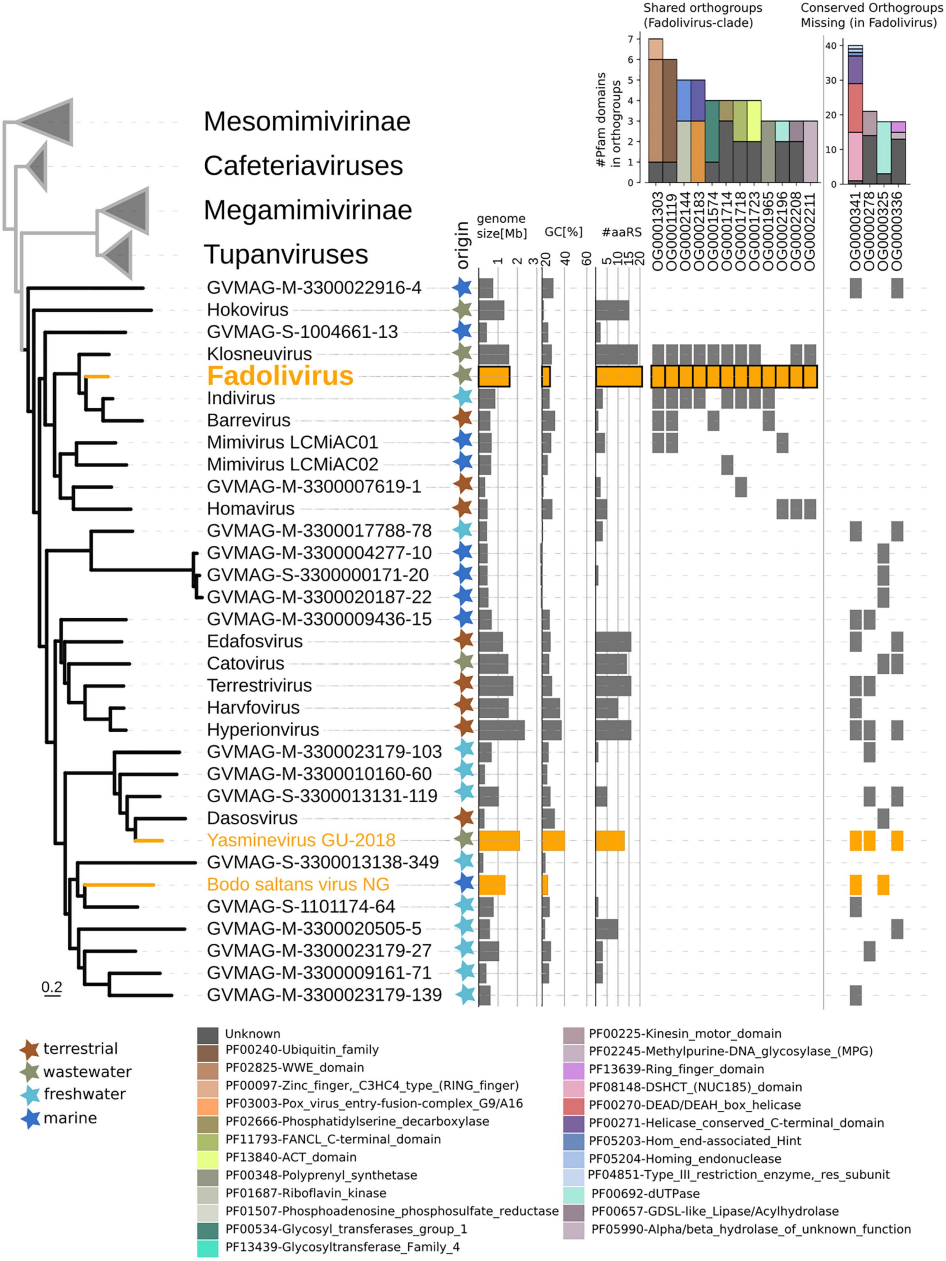
Phylogenetic position of Fadolivirus in the Mimiviridae and genome features. Maximum-likelihood phylogenetic tree (IQ-tree LG + F + R6) of the Mimiviridae inferred from a concatenated protein alignment of five core nucleocytoplasmic virus orthologous genes (NCVOGs; Yutin et al., 2009). Branches in orange represent viral isolates, and branches in black represent previously published metagenome-assembled genomes. The tree is rooted at the proposed viral subfamily Mesomimivirinae.Colored stars next to the tree indicate environmental origin. Bars next to the tree show genome features such as genome size, GC content and total number of proteins that contain aminoacyl-tRNA synthetase (aaRS)-related Pfam domain. Further, the presence/absence matrix next to the tree indicates protein families that are present in Fadolivirus and its closest relatives but absent in other members of the Mimiviridae; and protein families that are present in more than 30% of viral genomes in the Mimiviridae but not in Fadolivirus and its closest relatives. Stacked bars show the total count and annotation of pfam domains present in each of these protein families.

**Figure 6:**
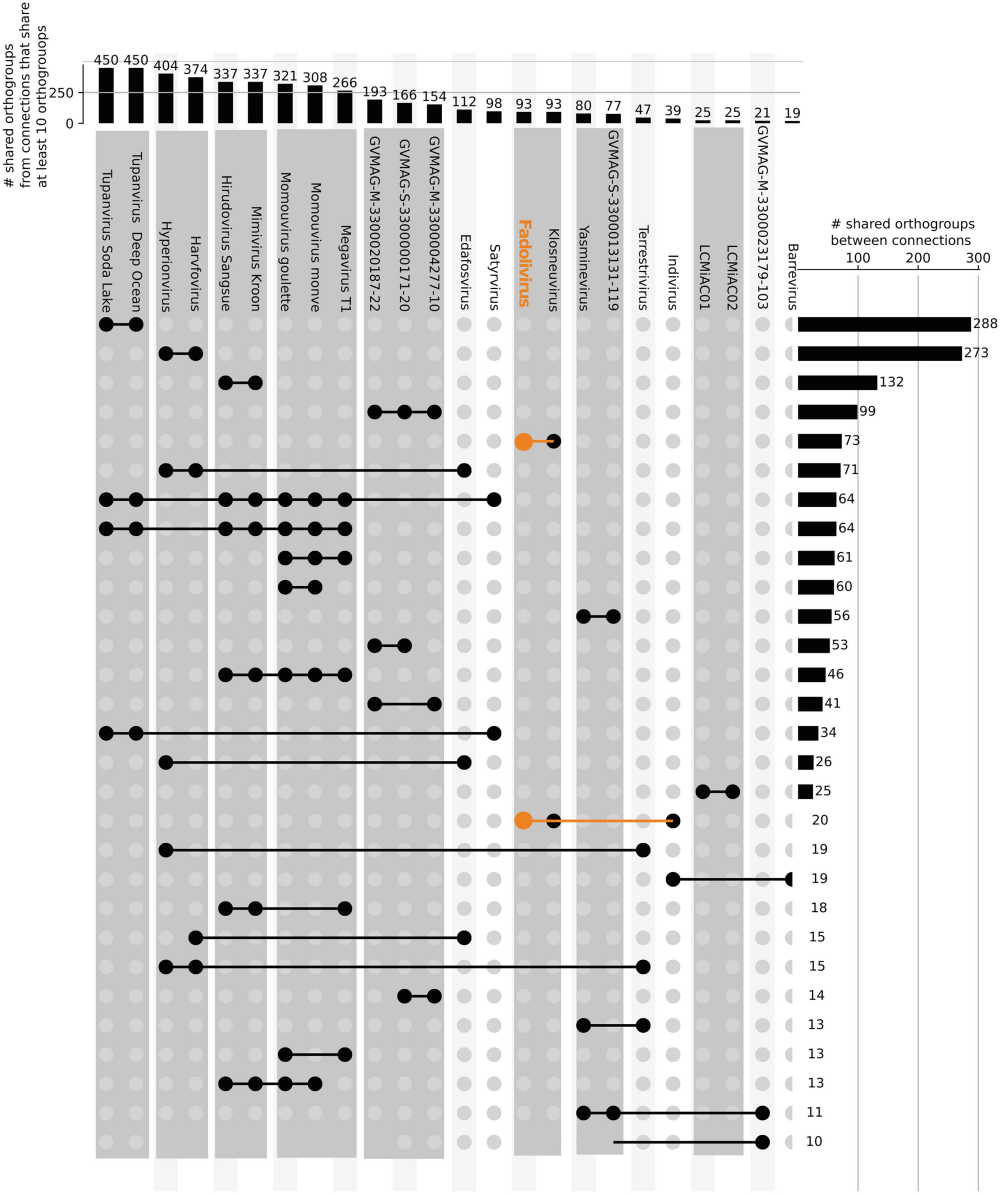
Orthologous genes shared by viruses in the *Klosneuvirinae* and some *Mimiviridae*. Connected lines highlight viruses sharing at least 10 orthologous genes. The bars at the top panel show the total number of shared orthologs between viruses that share at least 10 orthogroups. The bars in the right panel indicate the total number of shared orthogroups between visualized groups of viral genomes (connected by lines in the same row).

In the species tree of the Klosneuvirinae, Fadolivirus branched in a monophyletic clade with Klosneuvirus, Barrevirus and Indivirus (Figure 5). This result corresponded well with the complimentary best hit analysis described above. Genomes of members of this clade have a similar GC content of around 30% and genomes of Fadolivirus and Klosneuvirus express a similar genome size with approximately 1.5Mb, while Indivirus and Barrevirus have much smaller genomes of under 1Mb. Yasminevirus, another isolated member of the Klosneuvirinae branches divergent from Fadolivirus in the species tree and groups together with Dasosvirus. The third isolate of the Klosneuvirinae, Bodo saltans virus, groups in its own distinct clade. Genomic features and hallmarks that are found in Fadolivirus and Yasminevirus but not in Bodo saltans virus are aaRS. Clusters of orthologous proteins make it possible to observe significant diversity between Mimiviridae (Figure 6). There were 12 protein families that are almost exclusively found in the Klosneuvirus/Fadolivirus clade and 4 families that are widespread in the Klosneuvirinae but absent in members of the Klosneuvirus/Fadolivirus clade. Seventy-three orthologs are exclusively shared between Klosneuvirus and Fadolivirus; however, only 20 orthologs are exclusively shared between Fadolivirus, Klosneuvirus and Yasminevirus.

## Discussion

Fadolivirus is a new viral isolate with up to 1.59 Mbp, belonging to the sub-family Klosneuvirinae. Its replicative cycle in *V. vermiformis* is comparable to other NCLDVs that have been described, such as Yasminevirus (Bajrai et al., 2019). Fadolivirus shows significant proximity to Klosneuvirus KNV1, virus described by a previous metagenomic study (Schulz et al., 2017), by phylogenetic analysis and by genomic comparison. However, even though these metagenomes assembled viral genomes were of estimated high completeness and low contamination, their hosts and the capacity of each virus to have multiple hosts often remain elusive. Co-occurrence study in Tara Oceans tried to identify probable hosts but their analyses identified families of virus with their probable host in Eukaryotes such as the dominance of Mimiviridae in relation with Alveolata, Opisthokonta, Rhizaria, and Stramenopiles (Meng et al., 2021). Further, based on detection of horizontal gene transfer events in members of the Klosneuvirinae have been suggested to infect Anthoathecata, fungi, arthropods, and various protists including slime molds (Schulz et al., 2020). Indeed, at this time, among amoeba only Tupanviruses have been shown to infect various protists with a similar pattern in *A. castellanii* and in *V. vermiformis* (Abrahão et al., 2018; Silva et al., 2019). Fadolivirus, contrary to earlier predictions, seemed to be rather host specific as it was only able to replicate in *V. vermiformis* (strain CDC19) and not in the *Vermamoeba* strain chuv172. Fadolivirus was also unable to infect any of the other diverse protists that we used in our infection experiments. However, our data are limited to the 8 different protists that were used in the infection experiments. Fadolivirus presents a near complete translation system similar to the ones described in Klosneuvirus (Schulz et al., 2017) and Tupanvirus (Abrahão et al., 2018). Moreover, Fadolivirus has additional copies of tRNA synthetases (Asx, Glutaminyl and Ile-lysidine tRNAsynthetases). However, a histidine tRNA synthetase was not found in its genome. It remains unclear whether this absence could lead to a restricted host range that we observed for Fadolivirus. Further analyses will be necessary to provide a more comprehensive overview of potential hosts for this virus. In particular, virus-encoded transcription factors have been proven as being key proteins in members of the *Mimiviridae* during infection of their amoeba host (Bekliz et al., 2018). The presence of translation system genes in Mimiviruses, Tupanviruses, Cafeteria roenbergensis virus, and Klosneuvirinae including Yasminevirus and Bodo saltans virus and combined with the paucity of these genes in the common ancestor of these viruses highlights that such an expanded genetic complement was acquired multiple times and independently in a lineage-specific style of evolution (Schulz et al., 2017; Koonin and Yutin, 2018; Bäckström et al., 2019).

The surprising features of this virus reside in the presence of predicted subunits A and D of ATPase. The first description of vacuolar (H+)-ATPase highlights the close proximity of V and F-type of ATPase D in their catalytic function (Nelson et al., 1995). Recently, during a giant viral infection, it was shown that the shift of the carbonaceous biomass of the host concerned 6–17% for viral production, probably due to a Cedratvirus (Kördel et al., 2021). This observation underlined the fact that viral production requires a greater production of energy by the infected cells. Further, active glycolysis and tricarboxylic acid cycles were identified by metagenomic studies (Moniruzzaman et al., 2020a; Ha et al., 2021) and some proteins were also found in Pandoravirus (Aherfi et al., 2020) and could have a confirmed functional activity such as the isocitrate dehydrogenase protein. Concerning the predicted ATPase subunit A, it is also present in some Klosneuvirinae. We could hypothesize that the presence of a more complete system for energy generation system in the ancestor of the *Klosneuvirinae* indicates that the involved genes have been lost in some viral lineages, or those sequences could be highly divergent in ways that are currently not identified by our bioinformatics comparisons. However, for the subunit D this protein is only present for Fadolivirus and could have been acquired from eukaryotes. Nevertheless, in this study we did not perform transcriptomic and proteomic analyses on these putative ATPase subunits; this constitutes a limitation to demonstrate their functional activity.

Meanwhile, novel viruses have been described and appeared as smaller DNA viruses among NCLDV with high speciation to their hosts (Boratto et al., 2020; Subramaniam et al., 2020). Their descriptions bring new opportunities to understanding the diversity and evolution across *Nucleocytoviricota,* and energy systems genes such as the ones observed in Fadolivirus would appear to play a major role during infection by giant viruses.

## Data Availability Statement

The datasets presented in this study can be found in online repositories. The names of the repository/repositories and accession number(s) can be found in the article/Supplementary Material.

## Author Contributions

JA, FS, and BL designed the study and experiments. JA performed cytometry acquisition. JA and FDP performed electron microscopy. JA, FS, and AL performed bioinformatics analyses. JA, FS, TW, and BL wrote the manuscript. All authors approved the final manuscript.

## Funding

This work was supported by the French Government under the “Investissements d’avenir” (Investments for the Future) programme managed by the Agence Nationale de la Recherche (ANR, fr: National Agency for Research) (reference: Méditerranée Infection 10-IAHU-03). Parts of this study were performed by the US Department of Energy Joint Genome Institute, a DOE Office of Science User Facility and made use of resources of the National Energy Research Scientific Computing Center, both supported by the Office of Science of the US Department of Energy under Contract no. DE-AC02–05CH11231.

## Conflict of Interest

The authors declare that the research was conducted in the absence of any commercial or financial relationships that could be construed as a potential conflict of interest.

## Publisher’s Note

All claims expressed in this article are solely those of the authors and do not necessarily represent those of their affiliated organizations, or those of the publisher, the editors and the reviewers. Any product that may be evaluated in this article, or claim that may be made by its manufacturer, is not guaranteed or endorsed by the publisher.

## Footnotes

^1^ https://imagej.nih.gov/ij/

## References

[ref1] Abrahão J. Silva L. Silva L. S. Khalil J. Y. B. Rodrigues R. Arantes T.. (2018). Tailed giant Tupanvirus possesses the most complete translational apparatus of the known virosphere. Nat. Commun. 9, 1–12. doi: 10.1038/s41467-018-03168-1, PMID: 29487281 PMC5829246

[ref2] Aherfi S. Andreani J. Baptiste E. Oumessoum A. Dornas F. P. Andrade A. C. D. S. P.. (2018). A large open Pangenome and a small Core genome for Giant Pandoraviruses. Front. Microbiol. 9:1486. doi: 10.3389/fmicb.2018.01486, PMID: 30042742 PMC6048876

[ref3] Aherfi S. Belhaouari D. B. Pinault L. Baudoin J.-P. Decloquement P. Abrahão J.. (2020). Tricarboxylic acid cycle and proton gradient in Pandoravirus massiliensis: is it still a virus? bioRxiv [Preprint]. doi: 10.1101/2020.09.21.306415 PMC8857278 34556816

[ref4] Andreani J. Aherfi S. Bou Khalil J. Y. Di Pinto F. Bitam I. Raoult D.. (2016). Cedratvirus, a double-Cork structured Giant virus, is a distant relative of Pithoviruses. Viruses 8:300. doi: 10.3390/v8110300, PMID: 27827884 PMC5127014

[ref5] Andreani J. Bou Khalil J. Y. Sevvana M. Benamar S. Di Pinto F. Bitam I.. (2017). Pacmanvirus, a new giant icosahedral virus at the crossroads between Asfarviridae and Faustoviruses. J. Virol. 91, e00212–e00217. doi: 10.1128/JVI.00212-17 28446673 PMC5487549

[ref6] Andreani J. Khalil J. Y. B. Baptiste E. Hasni I. Michelle C. Raoult D.. (2018). Orpheovirus IHUMI-LCC2: a new virus among the Giant viruses. Front. Microbiol. 8:2643. doi: 10.3389/fmicb.2017.02643, PMID: 29403444 PMC5786535

[ref7] Antipov D. Korobeynikov A. McLean J. S. Pevzner P. A. (2016). hybridSPAdes: an algorithm for hybrid assembly of short and long reads. Bioinformatics 32, 1009–1015. doi: 10.1093/bioinformatics/btv688, PMID: 26589280 PMC4907386

[ref8] Antwerpen M. H. Georgi E. Zoeller L. Woelfel R. Stoecker K. Scheid P. (2015). Whole-genome sequencing of a pandoravirus isolated from keratitis-inducing acanthamoeba. Genome Announc. 3, e00136–e00115. doi: 10.1128/genomeA.00136-15, PMID: 25814595 PMC4384135

[ref9] Bäckström D. Yutin N. Jørgensen S. L. Dharamshi J. Homa F. Zaremba-Niedwiedzka K.. (2019). Virus genomes from Deep Sea sediments expand the ocean Megavirome and support independent origins of viral gigantism. MBio 10, e02497–e02418. doi: 10.1128/mBio.02497-18, PMID: 30837339 PMC6401483

[ref10] Bajrai L. H. Mougari S. Andreani J. Baptiste E. Delerce J. Raoult D.. (2019). Isolation of Yasminevirus, the first member of Klosneuvirinae isolated in coculture with Vermamoeba vermiformis, demonstrates an extended arsenal of translational apparatus components. J. Virol. 94, e01534–e01519. doi: 10.1128/JVI.01534-19 31597770 PMC6912108

[ref11] Balakrishna A. M. Basak S. Manimekalai M. S. S. Grüber G. (2015). Crystal structure of subunits D and F in complex gives insight into energy transmission of the eukaryotic V-ATPase from Saccharomyces cerevisiae. J. Biol. Chem. 290, 3183–3196. doi: 10.1074/jbc.M114.622688, PMID: 25505269 PMC4318993

[ref12] Barik S. (2018). A family of novel Cyclophilins, conserved in the Mimivirus genus of the Giant DNA viruses. Comput. Struct. Biotechnol. J. 16, 231–236. doi: 10.1016/j.csbj.2018.07.001, PMID: 30069285 PMC6068286

[ref13] Bekliz M. Azza S. Seligmann H. Decloquement P. Raoult D. La Scola B. (2018). Experimental analysis of Mimivirus translation initiation factor 4a reveals its importance in viral protein translation during infection of Acanthamoeba polyphaga. J. Virol. 92, e00337–e00318. doi: 10.1128/JVI.00337-18, PMID: 29514904 PMC5923065

[ref14] Bertelli C. Mueller L. Thomas V. Pillonel T. Jacquier N. Greub G. (2017). Cedratvirus lausannensis-digging into Pithoviridaediversity. Environ. Microbiol. 19, 4022–4034. doi: 10.1111/1462-2920.13813, PMID: 28618143

[ref15] Boratto P. V. M. Oliveira G. P. Machado T. B. Andrade A. C. S. P. Baudoin J.-P. Klose T.. (2020). Yaravirus: a novel 80-nm virus infecting Acanthamoeba castellanii. Proc. Natl. Acad. Sci. 7:202001637. doi: 10.1073/pnas.2001637117, PMID: 32601223 PMC7368276

[ref16] Boratyn G. M. Schäffer A. A. Agarwala R. Altschul S. F. Lipman D. J. Madden T. L. (2012). Domain enhanced lookup time accelerated BLAST. Biol. Direct 7, 12–14. doi: 10.1186/1745-6150-7-12, PMID: 22510480 PMC3438057

[ref17] Boudjemaa H. Andreani J. Bitam I. La Scola B. (2020). Diversity of amoeba-associated Giant viruses isolated in Algeria. Diversity 12:215. doi: 10.3390/d12060215

[ref18] Capella-Gutiérrez S. Silla-Martínez J. M. Gabaldón T. (2009). trimAl: a tool for automated alignment trimming in large-scale phylogenetic analyses. Bioinformatics 25, 1972–1973. doi: 10.1093/bioinformatics/btp348, PMID: 19505945 PMC2712344

[ref19] Chamizo-Ampudia A. Sanz-Luque E. Llamas A. Galvan A. Fernandez E. (2017). Nitrate reductase regulates plant nitric oxide homeostasis. Trends Plant Sci. 22, 163–174. doi: 10.1016/j.tplants.2016.12.001, PMID: 28065651

[ref20] Colson P. La Scola B. Levasseur A. Caetano-Anollés G. Raoult D. (2017). Mimivirus: leading the way in the discovery of giant viruses of amoebae. Nat. Rev. Microbiol. 15, 243–254. doi: 10.1038/nrmicro.2016.197, PMID: 28239153 PMC7096837

[ref21] Darling A. C. E. Mau B. Blattner F. R. Perna N. T. (2004). Mauve: multiple alignment of conserved genomic sequence with rearrangements. Genome Res. 14, 1394–1403. doi: 10.1101/gr.2289704, PMID: 15231754 PMC442156

[ref22] Deeg C. M. Chow C.-E. T. Suttle C. A. (2018). The kinetoplastid-infecting Bodo saltans virus (BsV), a window into the most abundant giant viruses in the sea. elife 7:e33014. doi: 10.7554/eLife.33014, PMID: 29582753 PMC5871332

[ref23] Edgar R. C. (2004). MUSCLE: a multiple sequence alignment method with reduced time and space complexity. BMC Bioinfo. 5:113. doi: 10.1186/1471-2105-5-113, PMID: 15318951 PMC517706

[ref24] El-Gebali S. Mistry J. Bateman A. Eddy S. R. Luciani A. Potter S. C.. (2019). The Pfam protein families database in 2019. Nucl. Acids Res. 47, D427–D432. doi: 10.1093/nar/gky995, PMID: 30357350 PMC6324024

[ref25] Emms D. M. Kelly S. (2015). OrthoFinder: solving fundamental biases in whole genome comparisons dramatically improves orthogroup inference accuracy. Genome Biol. 16, 1–14. doi: 10.1186/s13059-015-0721-2, PMID: 26243257 PMC4531804

[ref26] Forterre P. (2016). To be or not to be alive: how recent discoveries challenge the traditional definitions of viruses and life. Stud. Hist. Philos. Sci. Part C: Stud. Hist. Philos. Biol. Biomed. Sci. 59, 100–108. doi: 10.1016/j.shpsc.2016.02.013 26996409

[ref27] Gaia M. Benamar S. Boughalmi M. Pagnier I. Croce O. Colson P.. (2014). Zamilon, a novel Virophage with Mimiviridae host specificity. PLoS One 9:e94923. doi: 10.1371/journal.pone.0094923, PMID: 24747414 PMC3991649

[ref28] Geballa-Koukoulas K. Andreani J. La Scola B. Blanc G. (2021). The Kaumoebavirus LCC10 genome reveals a unique Gene Strand bias among "extended Asfarviridae". Viruses 13:148. doi: 10.3390/v13020148, PMID: 33498382 PMC7909422

[ref29] Geballa-Koukoulas K. Boudjemaa H. Andreani J. La Scola B. Blanc G. (2020). Comparative genomics unveils regionalized evolution of the Faustovirus genomes. Viruses 12:577. doi: 10.3390/v12050577, PMID: 32456325 PMC7290515

[ref30] Guy L. Kultima J. R. Andersson S. G. E. (2010). genoPlotR: comparative gene and genome visualization in R. Bioinformatics 26, 2334–2335. doi: 10.1093/bioinformatics/btq413, PMID: 20624783 PMC2935412

[ref31] Ha A. D. Moniruzzaman M. Aylward F. O. (2021). High transcriptional activity and diverse functional repertoires of hundreds of Giant viruses in a coastal marine system. bioRxiv 2021:e0029321. doi: 10.1101/2021.03.08.434518, PMID: 34254826 PMC8407384

[ref32] Hyatt D. Chen G.-L. LoCascio P. F. Land M. L. Larimer F. W. Hauser L. J. (2010). Prodigal: prokaryotic gene recognition and translation initiation site identification. BMC Bioinformatics 11:119. doi: 10.1186/1471-2105-11-119, PMID: 20211023 PMC2848648

[ref33] Jones P. Binns D. Chang H.-Y. Fraser M. Li W. McAnulla C.. (2014). InterProScan 5: genome-scale protein function classification. Bioinformatics 30, 1236–1240. doi: 10.1093/bioinformatics/btu031, PMID: 24451626 PMC3998142

[ref34] Katoh K. Standley D. M. (2016). A simple method to control over-alignment in the MAFFT multiple sequence alignment program. Bioinformatics 32, 1933–1942. doi: 10.1093/bioinformatics/btw108, PMID: 27153688 PMC4920119

[ref35] Kelley L. A. Mezulis S. Yates C. M. Wass M. N. Sternberg M. J. E. (2015). The Phyre2 web portal for protein modeling, prediction and analysis. Nat. Protoc. 10, 845–858. doi: 10.1038/nprot.2015.053, PMID: 25950237 PMC5298202

[ref36] Khalil J. Y. B. Robert S. Reteno D. G. Andreani J. Raoult D. La Scola B. (2016). High-throughput isolation of Giant viruses in liquid medium using automated flow cytometry and fluorescence staining. Front. Microbiol. 7:26. doi: 10.3389/fmicb.2016.00026, PMID: 26858703 PMC4731542

[ref37] Koonin E. V. Dolja V. V. Krupovic M. Varsani A. Wolf Y. I. Yutin N.. (2020). Global organization and proposed Megataxonomy of the virus world. Microbiol. Mol. Biol. Rev. 84, e00061–e00019. doi: 10.1128/MMBR.00061-19, PMID: 32132243 PMC7062200

[ref38] Koonin E. V. Yutin N. (2010). Origin and evolution of eukaryotic large nucleo-cytoplasmic DNA viruses. Intervirology 53, 284–292. doi: 10.1159/000312913, PMID: 20551680 PMC2895762

[ref39] Koonin E. V. Yutin N. (2018). Multiple evolutionary origins of giant viruses. F1000Research 7:1840. doi: 10.12688/f1000research.16248.1 PMC6259494 30542614

[ref40] Kördel M. Svenda M. Reddy H. K. N. Fogelqvist E. Arsana K. G. Y. Hamawandi B.. (2021). Quantitative conversion of biomass in giant DNA virus infection. Sci. Rep. 11, 1–12. doi: 10.1038/s41598-021-83547-9, PMID: 33658544 PMC7930090

[ref41] La Scola B. (2003). A Giant Virus in Amoebae. Science 299:2033. doi: 10.1126/science.1081867, PMID: 12663918

[ref42] La Scola B. Desnues C. Pagnier I. Robert C. Barrassi L. Fournous G.. (2008). The virophage as a unique parasite of the giant mimivirus. Nature 455, 100–104. doi: 10.1038/nature07218, PMID: 18690211

[ref43] Lamb D. C. Follmer A. H. Goldstone J. V. Nelson D. R. Warrilow A. G. Price C. L.. (2019). On the occurrence of cytochrome P450 in viruses. Proc. Natl. Acad. Sci. 116, 12343–12352. doi: 10.1073/pnas.1901080116, PMID: 31167942 PMC6589655

[ref44] Legendre M. Bartoli J. Shmakova L. Jeudy S. Labadie K. Adrait A.. (2014). Thirty-thousand-year-old distant relative of giant icosahedral DNA viruses with a pandoravirus morphology. Proc. Natl. Acad. Sci. 111, 4274–4279. doi: 10.1073/pnas.1320670111, PMID: 24591590 PMC3964051

[ref45] Legendre M. Fabre E. Poirot O. Jeudy S. Lartigue A. Alempic J.-M.. (2018). Diversity and evolution of the emerging Pandoraviridae family. Nat. Commun. 9, 1–12. doi: 10.1038/s41467-018-04698-4 29891839 PMC5995976

[ref46] Letunic I. Bork P. (2016). Interactive tree of life (iTOL) v3: an online tool for the display and annotation of phylogenetic and other trees. Nucl. Acids Res. 44, W242–W245. doi: 10.1093/nar/gkw290, PMID: 27095192 PMC4987883

[ref47] Levasseur A. Andreani J. Delerce J. Bou Khalil J. Robert C. La Scola B.. (2016). Comparison of a modern and fossil Pithovirus reveals its genetic conservation and evolution. Genome Biol. Evol. 8, 2333–2339. doi: 10.1093/gbe/evw153 27389688 PMC5010891

[ref48] Lowe T. M. Chan P. P. (2016). tRNAscan-SE on-line: integrating search and context for analysis of transfer RNA genes. Nucl. Acids Res. 44, W54–W57. doi: 10.1093/nar/gkw413, PMID: 27174935 PMC4987944

[ref49] Marchler-Bauer A. Bryant S. H. (2004). CD-search: protein domain annotations on the fly. Nucleic Acids Res. 32, W327–W331. doi: 10.1093/nar/gkh454, PMID: 15215404 PMC441592

[ref50] Meng L. Endo H. Blanc-Mathieu R. Chaffron S. Hernández-Velázquez R. Kaneko H.. (2021). Quantitative assessment of nucleocytoplasmic large DNA virus and host interactions predicted by co-occurrence analyses. mSphere 6, e01298–e01220. doi: 10.1128/mSphere.01298-20, PMID: 33883262 PMC8546719

[ref51] Moniruzzaman M. Martinez-Gutierrez C. A. Weinheimer A. R. Aylward F. O. (2020a). Dynamic genome evolution and complex virocell metabolism of globally-distributed giant viruses. Nat. Commun. 11, 1710–1711. doi: 10.1038/s41467-020-15507-2, PMID: 32249765 PMC7136201

[ref52] Moniruzzaman M. Weinheimer A. R. Martinez-Gutierrez C. A. Aylward F. O. (2020b). Widespread endogenization of giant viruses shapes genomes of green algae. Nature 588, 141–145. doi: 10.1038/s41586-020-2924-2, PMID: 33208937

[ref53] Nelson H. Mandiyan S. Nelson N. (1995). A bovine cDNA and a yeast gene (VMA8) encoding the subunit D of the vacuolar H(+)-ATPase. Proc. Natl. Acad. Sci. 92, 497–501. doi: 10.1073/pnas.92.2.497 7831318 PMC42768

[ref54] Nguyen L.-T. Schmidt H. A. Haeseler von A. Minh B. Q. (2015). IQ-TREE: a fast and effective stochastic algorithm for estimating maximum-likelihood phylogenies. Mol. Biol. Evol. 32, 268–274. doi: 10.1093/molbev/msu300, PMID: 25371430 PMC4271533

[ref55] Philippe N. Legendre M. Doutre G. Couté Y. Poirot O. Lescot M.. (2013). Pandoraviruses: amoeba viruses with genomes up to 2.5 Mb reaching that of parasitic eukaryotes. Science 341, 281–286. doi: 10.1126/science.1239181, PMID: 23869018

[ref56] Reteno D. G. Benamar S. Khalil J. B. Andreani J. Armstrong N. Klose T.. (2015). Faustovirus, an asfarvirus-related new lineage of giant viruses infecting amoebae. J. Virol. 89, 6585–6594. doi: 10.1128/JVI.00115-15, PMID: 25878099 PMC4468488

[ref57] Rodrigues R. A. L. Andreani J. Andrade A. C. D. S. P. Machado T. B. Abdi S. Levasseur A.. (2018a). Morphologic and genomic analyses of new isolates reveal a second lineage of cedratviruses. J. Virol. 92, 00372–00318. doi: 10.1128/JVI.00372-18, PMID: 29695424 PMC6002711

[ref58] Rodrigues R. A. L. Mougari S. Colson P. La Scola B. Abrahão J. S. (2018b). “Tupanvirus,” a new genus in the family Mimiviridae. Arch. Virol. 15, 243–247. doi: 10.1007/s00705-018-4067-4, PMID: 30291500

[ref59] Schulz F. Alteio L. Goudeau D. Ryan E. M. Yu F. B. Malmstrom R. R.. (2018). Hidden diversity of soil giant viruses. Nat. Commun. 9, 1–9. doi: 10.1038/s41467-018-07335-2, PMID: 30451857 PMC6243002

[ref60] Schulz F. Roux S. Paez-Espino D. Jungbluth S. Walsh D. A. Denef V. J.. (2020). Giant virus diversity and host interactions through global metagenomics. Nature 578, 432–436. doi: 10.1038/s41586-020-1957-x, PMID: 31968354 PMC7162819

[ref61] Schulz F. Yutin N. Ivanova N. N. Ortega D. R. Lee T. K. Vierheilig J.. (2017). Giant viruses with an expanded complement of translation system components. Science 356, 82–85. doi: 10.1126/science.aal4657, PMID: 28386012

[ref62] Shinn G. L. Bullard B. L. (2018). Ultrastructure of Meelsvirus: a nuclear virus of arrow worms (phylum Chaetognatha) producing giant “tailed” virions. PLoS One 13:e0203282. doi: 10.1371/journal.pone.0203282, PMID: 30231047 PMC6145532

[ref63] Silva L. K. D. S. Andrade A. C. D. S. P. Dornas F. P. Rodrigues R. A. L. Arantes T. Kroon E. G.. (2018). Cedratvirus getuliensis replication cycle: an in-depth morphological analysis. Sci. Rep. 8, 1–11. doi: 10.1038/s41598-018-22398-3, PMID: 29507337 PMC5838162

[ref64] Silva L. C. F. Rodrigues R. A. L. Oliveira G. P. Dornas F. P. La Scola B. Kroon E. G.. (2019). Microscopic analysis of the Tupanvirus cycle in Vermamoeba vermiformis. Front. Microbiol. 10:671. doi: 10.3389/fmicb.2019.00671, PMID: 31001237 PMC6456662

[ref65] Subramaniam K. Behringer D. C. Bojko J. Yutin N. Clark A. S. Bateman K. S.. (2020). A new family of DNA viruses causing disease in crustaceans from diverse aquatic biomes. MBio 11, e02938–e02919. doi: 10.1128/mBio.02938-19, PMID: 31937645 PMC6960288

[ref66] Suzan-Monti M. La Scola B. Barrassi L. Espinosa L. Raoult D. (2007). Ultrastructural characterization of the giant volcano-like virus factory of Acanthamoeba polyphaga Mimivirus. PLoS One 2:e328. doi: 10.1371/journal.pone.0000328, PMID: 17389919 PMC1828621

[ref67] Thai V. Renesto P. Fowler C. A. Brown D. J. Davis T. Gu W.. (2008). Structural, biochemical, and in vivo characterization of the first virally encoded cyclophilin from the Mimivirus. J. Mol. Biol. 378, 71–86. doi: 10.1016/j.jmb.2007.08.051, PMID: 18342330 PMC2884007

[ref68] Verneau J. Levasseur A. Raoult D. La Scola B. Colson P. (2016). MG-digger: an automated pipeline to search for Giant virus-related sequences in metagenomes. Front. Microbiol. 7:428. doi: 10.3389/fmicb.2016.00428, PMID: 27065984 PMC4814491

[ref69] Wilson W. H. Gilg I. C. Moniruzzaman M. Field E. K. Koren S. LeCleir G. R.. (2017). Genomic exploration of individual giant ocean viruses. ISME J. 15, 1736–1745. doi: 10.1038/ismej.2017.61 PMC5520044 28498373

[ref70] Yutin N. Wolf Y. I. Raoult D. Koonin E. V. (2009). Eukaryotic large nucleo-cytoplasmic DNA viruses: clusters of orthologous genes and reconstruction of viral genome evolution. Virol. J. 6:223. doi: 10.1186/1743-422X-6-223, PMID: 20017929 PMC2806869

[ref71] Zhao J. Benlekbir S. Rubinstein J. L. (2015). Electron cryomicroscopy observation of rotational states in a eukaryotic V-ATPase. Nature 521, 241–245. doi: 10.1038/nature14365, PMID: 25971514

